# An Efficient *Agrobacterium*-Mediated Genetic Transformation System for Gene Editing in Strawberry (*Fragaria* × *ananassa*)

**DOI:** 10.3390/plants13050563

**Published:** 2024-02-20

**Authors:** Fatema Akter, Suting Wu, Md Shariful Islam, Htin Kyaw, Jinwen Yang, Mingyue Li, Yuxin Fu, Jinxia Wu

**Affiliations:** Biotechnology Research Institute, Chinese Academy of Agricultural Sciences, Beijing 100081, China; fatemasau4058@gmail.com (F.A.); 82101201609@caas.cn (S.W.); sharifulcaas@gmail.com (M.S.I.); htinkyawyau@gmail.com (H.K.); jwyang0737@foxmail.com (J.Y.); limingyue0032@163.com (M.L.); jnsazn@163.com (Y.F.)

**Keywords:** *Fragaria* × *ananassa*, CRISPR/Cas9, callus induction, plant regeneration, explant, plant growth regulator

## Abstract

The octoploid-cultivated strawberry variety Benihope (*Fragaria* × *ananassa* Duch cv. Benihope) is an important commercial plant. It is highly susceptible to different diseases, which ultimately leads to a reduction in yield. Gene-editing methods, such as CRISPR/Cas9, demonstrate potential for improving disease resistance in the strawberry cv. Benihope. Establishing a plant regeneration system suitable for CRISPR/Cas9 gene editing is crucial for obtaining transgenic plants on a large scale. This research established a callus induction and plant regeneration system for *Agrobacterium*-mediated CRISPR/Cas9 gene editing in strawberry cv. Benihope by evaluating multiple types of explants and various plant growth regulators throughout the entire tissue culture process. The results showed that the efficiency of callus induction is strongly influenced by the type of explant and is highly sensitive to the combination of plant growth regulators. Among the different plant growth regulators employed, thidiazuron (TDZ), in combination with 2,4-dichlorophenoxyacetic acid (2,4-D), effectively induced callus formation and plant regeneration from explants derived from nutrient tissues such as runner tips and crowns. In addition, the regeneration experiment demonstrated that the addition of polyvinylpyrrolidone (PVPP) to the shoot regeneration medium could inhibit tissue browning. The gene-edited plants in which some or all of the *Fvb7-1*, *Fvb7-2*, *Fvb7-3*, and *Fvb7-4* genes in the MLO (*Mildew resistance Locus O*) gene family were knocked out by CRISPR/Cas9 system were obtained by applying the plant regeneration system developed in this study.

## 1. Introduction

The cultivated strawberry species, *Fragaria* × *ananassa* Duch., is one of the most important berry crops in the world and is vital for regional economies in many countries, such as China, USA, Mexico, Turkey, and Spain. It is an octoploid variety (8x = 2n = 56) that originated from the hybridization of the South American species *F. chiolensis* and the North American species *F. virginiana*. The modern strawberry variety, Benihope (*Fragaria* × *ananassa* Duch. cv. Benihope), was collected from Japan and commercially cultivated in China. More than 50% of the total area dedicated to strawberry cultivation is covered by this variety in China, particularly in Jiangsu and Zhejiang provinces. Benihope exhibits essential features, including excellent fruit shape, sweet taste, and high-yield performance, making it suitable for commercial cultivation. However, it is highly susceptible to many fungal diseases, such as powdery mildew, anthracnose, and gray mold [1].

Nowadays, gene-editing technology has great potential for improving existing varieties or generating new ones. Selecting specific genes of interest and applying them to strawberry gene engineering by gene-editing technology may be an effective method to improve strawberry disease resistance. Among the various gene-editing strategies, studies on the Clustered Regularly Interspaced Short Palindromic Repeats (CRISPR)/CRISPR-associated protein 9 (Cas9) system, used for target genome editing in different species, such as Arabidopsis [2], tobacco [3], rice [4], maize [5], and barley [6], have shown its broad application prospects in plant genome editing. Gene-editing technology has also been applied to strawberries in recent years, showing its significant role in improving strawberry varieties. Strawberry varieties of forest strawberries with high phosphorus use efficiency were selected by knocking out the *FvPHO2* gene [7]. Another example of gene-editing technology employed in diploid strawberries was the fine regulation of sugar content in strawberry fruit by an efficient cytosine-base editing system [8]. Although the application of gene editing in octoploid strawberries is rarely reported, a recent study on *FaTM6* gene editing in octoploid strawberries showed the key role of this gene in strawberry anther development [9].

The acquisition of transgenic plants carrying CRISPR/Cas9 mediated vectors is a critical step in gene editing using the CRISPR/Cas9 system. The *Agrobacterium*-mediated method has become more popular due to its low transgene copy number, cost-effectiveness, and enhanced impact on the target gene. An efficient transformation and regeneration system is of utmost importance for efficiently recovering transformed cells after *agro*-infection [10], which is a foundation for the successful implementation of *Agrobacterium*-mediated genetic transformation in vegetatively propagated plants such as strawberries. Competent methods for shoot organogenesis and regeneration could effectively improve the induction of shoot organogenesis from transformed cells, thereby increasing the probability of successfully obtaining CRISPR/Cas9-transgenic plants [11].

Explant type and treatment with growth regulators are the main factors affecting plant regeneration and, ultimately, the efficiency of genetic transformation. In strawberry regeneration, different types of explants have been assessed. Leaf discs [12,13,14,15,16,17] and petioles [13,16,18,19,20,21,22] provided the highest regeneration efficiency as explants. The regeneration rate of leaf discs could reach 85–90% [13,17]. It has been reported that petiole explants could sometimes achieve a higher regeneration efficiency compared with leaf and stipule explants [23,24]. Except for leaf discs and petioles, meristems obtained from the base of in vitro-grown plants could also bring a regeneration rate of 58.5% [25]. In addition, runners [26], roots [13,27], anthers [28], embryos [29], sepals [30], and protoplasts [31] have been used as explants for strawberry regeneration in limited research. In contrast, using stems, peduncles, stolons, and stipules as explants in strawberry regeneration resulted in regeneration rates below 7% [18,27,32,33].

On the other hand, various growth regulators also play vital roles in strawberry regeneration. Plant growth regulators, including auxin and cytokinin, are essential for meristematic tissue growth, cell division, cell differentiation, and the promotion of tissue culture processes. Auxins, such as indole-3-acetic acid (IAA), 1-naphthalene acetic acid (NAA), and indolyl-3-butyric acid (IBA), regulate cell growth, division, and elongation, while cytokinins, such as 6-benzylaminopurine (6-BA) and kinetin (KT), are involved in regulating various plant developmental processes, including shoot formation and root development. Different combinations of auxin and cytokinin concentrations could determine the direction of organogenesis. In strawberry tissue culture, different growth regulators and concentration combinations are required for different explants and regeneration stages, such as callus initiation, shoot regeneration, and rooting [34]. As a typical auxin, NAA has been widely used to induce callus formation in strawberry tissue culture. The use of 4 mg/L NAA or the combinations of 0.5 mg/L 6-BA and 0.75 mg/L NAA have been reported as effective in callus induction from leaf explants [35,36]. Treating leaf and runner tip explants with 4.0 mg/L NAA and 1.5 mg/L IBA also resulted in a high yield of callus [37]. Both NAA [38,39] and IBA [40,41,42,43,44] are primarily used for rooting in the strawberry regeneration system. For shoot regeneration after callus induction, 6-BA, a classical cytokinin, affects plant growth and development by stimulating cell division. 6-BA and IBA are generally added to the Murashige and Skoog (MS) medium [45] for the regeneration of cultivated strawberries [13,46,47,48]. The combination of 2.25 mg/L 6-BA and 0.5 mg/L IBA has been reported as the most satisfactory for achieving callus induction from strawberry (*Fragaria* × *ananassa* Duch.) stipules [27]. The effectiveness of combinations of auxin IAA, NAA, and cytokinin KT with 6-BA in shoot regeneration has also been evaluated [12,26,35,38,40,41,42,43,49]. In addition, gibberellin (GA3) and some chemical analogs, such as 2,4-D (2,4-dichlorophenoxyacetic acid), also play crucial roles in strawberry tissue culture.

TDZ (thidiazuron), a cytokinin-like plant growth regulator with intense activity on cell division, has been used alone or in synergy with other plant hormones in plant tissue culture for plant development [50]. The role of TDZ in morphogenesis is closely related to the metabolism of endogenous hormones, and TDZ sometimes acts not only as a cytokinin but also as an auxin, gibberellin, and ethylene [51]. TDZ, along with other plant growth regulators, such as 2,4-D, NAA, and 6-BA, has been used for callus induction in various species [52,53,54]. TDZ was also particularly effective in shoot induction from leaves, sepals [13,14,15,17,55,56,57,58,59,60,61], and petioles [62,63]. Callus derived from leaf explants achieved a high shoot regeneration efficiency of 90% when treated with 2.0 mg/L TDZ and 0.2 mg/L IBA [64]. TDZ has been reported to provide specific responses that are highly dependent on the strawberry tissue type [13], but its effects on shoot regeneration from other explant types, except leaf, sepals, and petioles, are not well understood.

In addition, tissue blackening is another severe problem in strawberry tissue culture, associated with changes in protein status, amino acid content, ethylene content, and the accumulation of sucrose and starch [65]. Tissue blackening is caused by copper-containing oxidase enzymes, such as polyphenol oxidases like tyrosinases. These enzymes are synthesized and released under oxidative conditions after tissue wounding [66] and eventually cause growth inhibition or the death of explants. To solve this issue, many researchers have tested various phenol traps, such as activated charcoal, polyvinylpyrrolidone (PVPP), and antioxidants, to prevent tissue blackening. PVPP decreased the accumulation of peroxidase and reduced the presence of phenolic compounds in the explant [65,66].

The difficulties of strawberry tissue culture limited the development of the research on strawberry gene functions, resulting in the slow progress of modern strawberry breeding. The octoploid strawberry “Benihope” has high commercial value. However, its complex genome brings difficulties to gene function research and trait improvement by breeding. In this research, we aimed to provide an efficient protocol plant regeneration system for this cultivar and evaluate its potential for gene editing octoploid strawberries. We assessed the regeneration efficiency of different tissue explants in strawberry tissue culture by employing different combinations of growth regulators and concentrations using strawberry cv. Benihope and developed an efficient CRISPR/Cas9 genetic transformation system for strawberry. We successfully knocked out the *MLO* (*Mildew resistance Locus O*) gene, which is responsible for the susceptibility of strawberries to powdery mildew, using an *Agrobacterium*-mediated binary CRISPR/Cas9 vector. The knockout plants were obtained by applying the highly efficient genetic transformation system we constructed, thereby providing an effective strategy for breeding disease-resistant strawberries.

## 2. Results

### 2.1. Effects of Plant Hormones on Callus Induction from Different Benihope Explants

**Figure 1:**
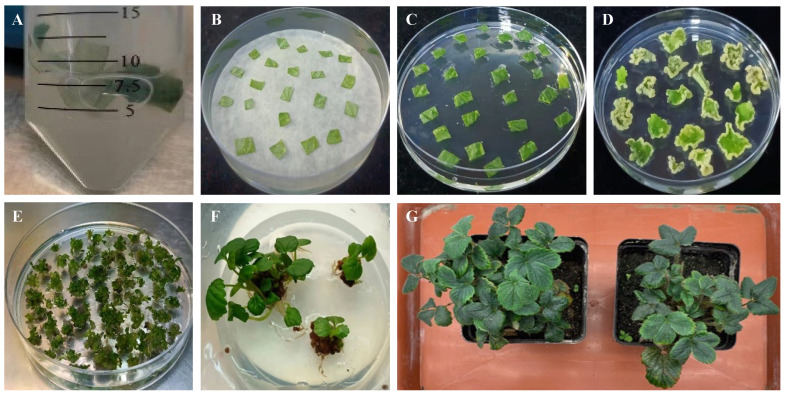
The regeneration process of *Agrobacterium*-mediated transgenic strawberry plants from leaf strip explants. (**A**) Leaf strip explants were infected with *Agrobacterium* in a liquid infection medium. (**B**) The infected leaf strips were placed on a co-cultivation medium with filter paper. (**C**) The leaf strips were moved to a callus induction medium to stimulate callus production. (**D**) After callus formation, the leaf strips were moved to a selection medium for resistance selection. (**E**) Surviving callus was transferred to a shoot induction and shoot elongation medium for shoot regeneration. (**F**) Seedlings were cultured on a seedling-strengthening medium. (**G**), *Agrobacterium*-mediated transgenic strawberry plants were cultivated in a climate chamber.

Since leaf strips (or discs) are the most commonly used explant for callus induction in strawberry tissue culture, we initially evaluated the effect of different hormone combinations in the medium on callus induction from leaf strip explants. The explants were infected with *Agrobacterium* carrying a CRISPR/Cas9 binary vector in a liquid infection medium and subsequently cultured on the co-cultivation medium for 3 days (Figure 1A,B). Then, during the selection process for callus induction and hygromycin-resistance selection, thirty different concentration combinations of growth regulators (6-BA + NAA, TDZ + 2,4-D, and TDZ + IBA) were tested using leaf strips of the “Benihope” cultivar as explants (Figure 1C,D; Table S1). The callus induction response to four different concentration combinations of 6-BA and NAA, three of TDZ and 2,4-D, and three of TDZ and IBA was tested. In general, the callus induction efficiency ranged from 5.3% to 90.0%. The combination of 0.5 mg/L 6-BA and 1.0 mg/L NAA treatment resulted in the highest callus induction efficiency of 90.0%. When the medium was treated with combinations of TDZ and 2,4-D, the overall callus induction rate was lower than that of the combinations of 6-BA and NAA but higher than that of the combinations of TDZ and IBA (Table 1). The combination of TDZ and IBA had the least significant effect on callus induction from leaf strips, resulting in only 5.3% (0.5 mg/L TDZ and 0.25 mg/L IBA) to 22.6% (0.5 mg/L TDZ and 0.1 mg/L IBA).

Then, the hormone combinations that callus induction from leaf strips positively responded to, excluding combinations of TDZ and IBA, which were found to have a low efficiency in inducing callus from leaves, were used to assess the efficiency of callus induction from other types of explants (Table 2 and Table S2). All explants exhibited a response to NAA at high concentrations (1–1.5 mg/L) when combined with 0.5 mg/L 6-BA, whereas only blended and petioles responded to NAA at low concentrations combined with 0.5 mg/L 6-BA. Hormone combinations of TDZ and 2,4-D showed a positive effect on runner tips and crowns, compared with the combinations of 6-BA and NAA. The combination of 0.5 mg/L TDZ and 0.25 mg/L 2,4-D obtained the highest efficiency of callus induction from blended runners (86.0%), runner tip meristematic tissues (72.3%), and crowns (87.3%).

Callus induction from seeds did not respond to combinations of 6-BA and NAA or TDZ and 2,4-D (Table 2). Therefore, we carried out experiments using three concentration combinations of 2,4-D and kinetin (KT) on seed explants. This approach was adopted based on previous reports demonstrating the efficacy of the 2,4-D and KT combination in inducing callus when using seeds as explants in tissue culture for various species [67,68,69]. Under the treatment of 2 mg/L 2,4-D and 0.5 mg/L KT, the callus-inducing efficiency of seeds reached its maximum value of 65.1%.

### 2.2. Callus Browning and PVPP

The callus pieces obtained from different types of explants after selection were collected and transferred to a medium supplemented with 38 different concentration combinations of plant growth regulators for shoot induction, including NAA, IAA, IBA, 6-BA, 2,4-D, and TDZ (Table S3). Upon the inoculation of the callus in the regeneration medium, all of the callus became black, and the medium turned brown or black within 3–4 days, thus hindering successful regeneration. The callus was frequently transferred to a fresh medium to prevent blackening but with meager success. Meanwhile, a portion of the callus was kept in the dark for 7 days, but it turned black within 2 days upon transfer into the light. 

To overcome this problem, PVPP was added to these concentration combinations. Shoot induction only responded to the treatment of 1 mg/L TDZ with 2 g/L PVPP when leaf strips (56.6%), runner tip meristematic tissues (35%), and crowns (13.3%) were used as explants, showing the successful mitigation of callus browning. However, blended leaves, petioles, and runner tips did not respond to any of the treatments, even when supplemented with PVPP in the medium. Instead, they either continued producing callus or exhibited browning on the shoot induction medium (Figure S3).

For shoot elongation, GA3 was commonly added to the medium with 2 g/L PVPP (Table 3 and Table S4). Shoot elongation did not occur in response to any of the three different concentrations of GA3 treatments (1–3 mg/L). When the shoot was treated with the combinations of 6-BA and IBA, shoot elongation was observed with a success rate of 50% for leaf strips (Figure 1E) and 23% for runner tip meristematic tissues as explants, using the treatment of 0.25 mg/L 6-BA with 0.2 mg/L IBA (Table 3). Although shoots elongated when other types of explants were used, they became brown and died rapidly within one week, resulting in a shoot elongation rate of 0% (as shown in Table 3).

### 2.3. Genotyping CRISPR/Cas9 Gene-Edited Plants

**Figure 2:**
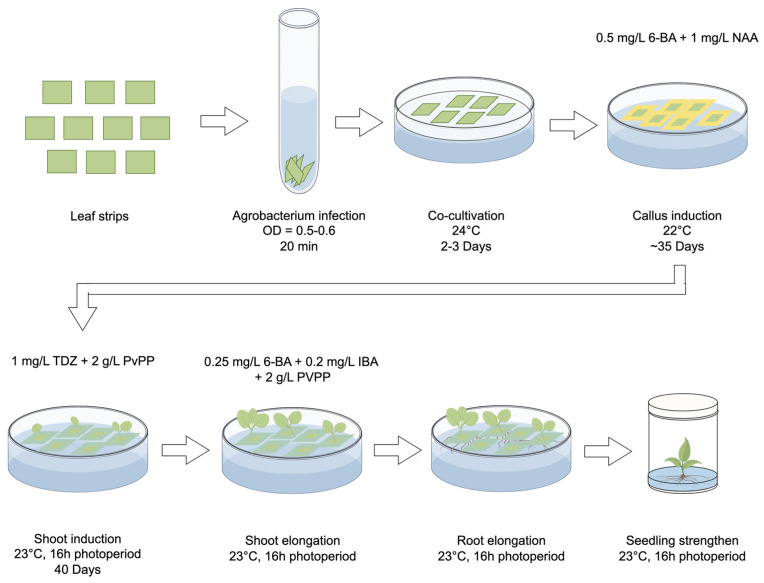
Workflow of the optimized plant generation system for the *Agrobacterium*-mediated transformation in strawberry. Leaf strips are identified as the most suitable explants for callus induction. After a 20 min infection with *Agrobacterium*, the leaf strips are placed on the co-cultivation medium under 24 °C for 2–3 days and then transferred to the callus induction medium and cultivated under 22 °C for ca. 35 days to induce callus formation. Subsequently, the callus is transferred to the shoot induction medium and cultured under 23 °C with a 16 h light/8 h dark photoperiod. After ca. 40 days, shoots begin to form and are then moved to the shoot elongation medium for several days. Larger shoots are then moved to grow on root elongation medium to initiate root formation. When roots become visible, the small seedlings can be planted in wide-mouth jars to strengthen their growth. The basic medium used for each step is listed in Method S1. Specific hormones added to each medium are demonstrated in this figure.

MS medium supplemented with 0.1 g/L IBA was used for root initiation (Figure 1F). Once the roots were well developed, the regenerated plants were transferred to soil and cultivated in a climate-controlled room with a 16 h light/8 h dark photoperiod at a temperature of 24 °C (Figure 1G). The optimized regeneration system, which utilized leaf strips as explants, is presented in Figure 2.

**Figure 3:**
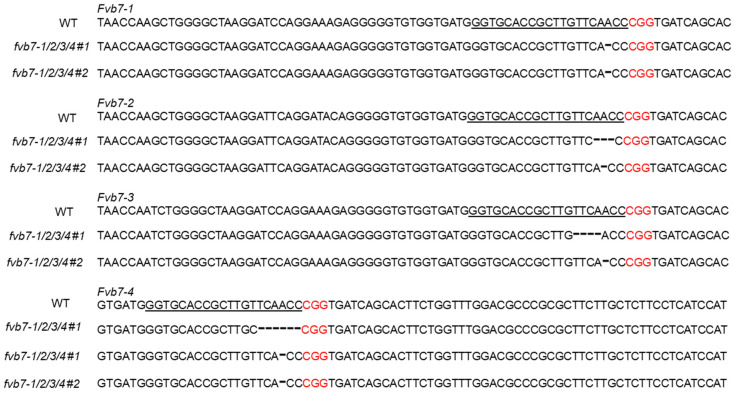
Hi-TOM assay of representative mutations around target regions of the *Fvb7-1/2/3/4* genes. The same gene-specific spacer sequence was selected within the exon of *Fvb7-1/2/3/4*. The chromatograms depict the *Fvb7-1/2/3/4* sequence in the wild type and the tetramutant *fvb7-1/2/3/4 #1* and *fvb7-1/2/3/4 #2* sequences. The target sequences are underlined, the protospacer adjacent motif (PAM) sequences are highlighted in red, and the dotted lines represent base deletions.

Twenty-six strawberry plants were successfully obtained through shoot regeneration using leaf segment explants, while nine plants were obtained using runner tip meristematic tissue explants. Callus produced by other explants failed to lead to plant formation during tissue culture. The genomic DNA of each regenerated plant was analyzed by PCR to verify the presence of the CRISPR/Cas9 sequence. Target fragments (572 bp) were amplified in Cas9p-positive plants, while no target fragments were amplified in Cas9p-negative and wild-type plants. Out of the twenty-six plants obtained from leaf explants, twenty-one were identified as positive transgenic plants and were further analyzed for the presence of expected sequence mutations. In contrast, only three out of the nine plants from runner tip meristematic tissue explants were positive. The twenty-four Cas9-positive plants were sequenced and analyzed using the Hi-TOM method (Table S5). Two out of the twenty-four plants showed no mutations. In the remaining twenty-two plants, a mutation in *Fvb7-1* was detected in twenty plants, with only two of them being single mutants of *Fvb7-1*. Eighteen plants carried base deletions within the targets of both *Fvb7-1* and *Fvb7-2* genes. Among these, thirteen plants were triple mutants, containing the mutation of the *Fvb7-3* gene, while two plants were tetramutants, with base deletions occurring within the targets of all four *Fvb7* genes (Figure 3). In addition, two out of the twenty-four Cas9-positive plants were identified as triple mutants, carrying mutations of *Fvb7-2*, *Fvb7-3*, and *Fvb7-4*. 

## 3. Discussion

Gene editing is now widely used in plant breeding and variety improvement. Thus, a stable and efficient plant tissue culture system is needed to achieve the successful transformation of gene-editing vectors and the generation of gene-edited plants. A precise balance of cytokinin and auxin is mandatory for facilitating cell division and organogenesis [70]. In our research, various types of explants were treated with different concentrations of NAA ranging from 0.5 to 1.5 mg/L with 6-BA, resulting in the production of callus in all explants except seeds (Table 1). The explants exhibited varied responses to NAA. Leaf-based explants, including leaf strips, blended leaves, and petioles, achieved their highest callus induction rate when treated with a combination of 1.0 mg/L NAA and 0.5 mg/L 6BA. In contrast, explants from runner tips and crowns resulted in shoot regeneration rather than callus formation under low NAA concentrations. In addition, a high concentration of 6-BA and a low concentration of NAA drastically inhibited callus formation in leaf strip explants (Table 1). This finding is consistent with the results reported by Husaini and Srivastava [48] for the strawberry cv. Chandler. 

TDZ is a phytohormone that has been underutilized in strawberry research, and its potential for strawberry tissue culture was thus investigated in this research. Combinations of TDZ and IBA were, in fact, employed as a cytokinin/auxin combination for callus induction. However, these combinations showed significantly lower callus induction efficiency in leaf explants compared with the combinations of TDZ and 2,4-D. All types of explants, except seeds, obtained a high callus induction rate when treated with 0.5 mg/L TDZ in combination with 0.25 mg/L 2,4-D. Cappelletti et al. [52] also reported a 100% callus induction rate from leaf explants of *Fragaria* × *ananassa* cv. Calypso when treated with a combination of 0.5 mg/L TDZ and 0.02 mg/L 2,4-D. In our experiments, when various types of strawberry explants were treated with different combinations of plant growth regulators, we found that NAA + 6-BA was the most efficient combination for callus induction in leaf-based explants (leaf strips, blended leaves, and petioles), whereas TDZ + 2,4-D was optimal for callus induction in runner tips, runner tip meristematic tissues, and crowns. These findings highlight that callus production efficiency is strongly affected by explant type and is highly sensitive to the combination of plant growth regulators; an improper combination can lead to low callus production. 

Seven different types of explants were tested to assess their callus induction efficiency (Figure S2). The highest callus induction efficiencies were recorded from leaf strips (90%), blended leaves (82.5%), petioles (88.3), blended runner tips (86%), runner tips meristematic tissues (72.3%), crowns (87.3%), and seeds (65.1%) under different treatments (Table 2). The type of explant has a great impact on plant regeneration via indirect shoot organogenesis, and various explant types showed significant differences in shoot regeneration capacity. In our research, shoot regeneration was observed only when leaf strips, runner tips meristematic tissues, and crowns were used as explants, with the highest shoot induction rate (56.6%) achieved using the leaf strip explant. Husaini and Srivastava [10] also reported that leaf discs had the highest regeneration rates across all strawberry cultivars. Therefore, leaf strips could be the preferred explant for strawberry transformation studies.

For shoot regeneration, the callus and the medium turned dark after the callus was transferred to the regular shoot induction medium. Madany and Parham [71] also observed a similar result during the tissue culture of *Fragaria* × *ananassa* Duch. cv. Camorosa and Selva. The darkening of the tissue is attributed to the browning of the medium, caused by the oxidation of the exuded phenolics from the excision of explants. This results in the obstruction of nutrient uptake from the medium, leading to the death of explants [72]. One way to minimize their exudation is by employing various absorbents and antioxidants. PVPP is a highly cross-linked form of polyvinylpyrrolidone (PVP), commonly used as a supplement to adsorb phenolics. Tang et al. [73] demonstrated the effectiveness of PVPP in inhibiting tissue necrosis in Virginia pine callus culture. Our results showed a similar result, namely that 2 g/L PVPP could overcome strawberry tissue blackening.

The development of gene-editing technology has great potential to accelerate crop improvement. As an important commercial fruit crop, the successful application of gene-editing technology in strawberries will be of great significance for strawberry variety improvement [7,8,9]. In this research, the CRISPR/Cas9 technology was applied to the octoploid strawberry “Benihope”. Gene-editing outcomes of the *Fvb7-1*, *Fvb7-2*, *Fvb7-3*, and *Fvb7-4* genes located on the seventh chromosome set in “Benihope” were evaluated. Each allelic target site should be sequenced to determine the gene-editing results of the transgenic plants at the genome level. However, the octoploid strawberry genome contains a large number of genes in the *MLO* gene family that exhibit high sequence similarities, making it challenging to edit all the *MLO* homologs functioning in powdery mildew susceptibility. Our results demonstrate that all four target genes were successfully edited in only two out of the twenty-four CRISPR/Cas9-positive plants. Therefore, further research on gene-editing system development should be conducted, given the complexity of the octoploid strawberry genome.

Meanwhile, the large-scale cultivation and successful acquisition of gene-edited plants remain significant bottlenecks in applying this technology to plant breeding. The editing efficiency of the CRISPR/Cas system has currently been a subject of extensive concern, but there are limited reports on tissue culture systems for CRISPR/Cas gene-edited plants. In our study, we successfully obtained gene-edited strawberry plants by introducing a CRISPR/Cas9-mediated gene-editing vector into strawberries through the *Agrobacterium*-mediated transformation method. However, in the tissue culture process, a large amount of callus was lost, resulting in low cultivation efficiency despite the achievement of a high callus induction rate of up to 90%. Most of the successfully induced callus died due to browning during the shoot regeneration process. Although the use of a PVPP-treated medium could increase the shoot survival rate, the shoot regeneration efficiency remained insufficient. Therefore, there is a need for continued development of culture medium formulations and cultivation conditions that prevent tissue browning and lead to improved efficiency of tissue culture for gene-edited plants.

In summary, we established a tissue culture system for obtaining *Agrobacterium*-mediated CRISPR/Cas9 gene-edited plants of the strawberry cultivar Benihope (*Fragaria* × *ananassa* Duch cv. Benihope). We achieved successful gene editing on all four alleles located on the same chromosome of the octoploid strawberry. Leaf strips were determined to be the most suitable option for callus induction after *Agrobacterium* infection. A combination of 0.5 mg/L 6-BA and 1 mg/L NAA was shown as the most efficient treatment for callus induction during the selection stage. Then, the callus was moved to a medium supplemented with 1 mg/L TDZ for shoot induction and a medium supplemented with a treatment combination of 0.25 mg/L 6-BA and 0.2 mg/L IBA for shoot elongation. Each of the media used during shoot regeneration required the addition of 2 g/L PVPP to suppress tissue browning. The seedlings regenerated on the shoot elongation medium could be moved to a seedling-strengthening medium to obtain stronger seedlings, which were then planted in pots and cultivated in a climate chamber for further applications. 

## 4. Materials and Methods

### 4.1. Plant Materials

To cultivate aseptic strawberry seedlings and conduct tissue culture, seeds and runner tips of the *Fragaria* × *ananassa* Duch. cv. Benihope were collected from strawberry plants grown in a greenhouse in Langfang, Hebei Province, China. The seeds were surface-sterilized using chlorine gas in a closed bell jar desiccator [74]. Three small Petri dishes containing mature strawberry seeds and one beaker with 100 mL of bleach and 3.5 mL of concentrated (12 N) HCL were placed inside the desiccator. The seeds were sterilized by the volatile gas for 16 h and then moved to a laminar flow cabinet for drying. Half of the sterilized seeds were used for callus induction, while the remaining seeds were germinated on a 1/2 MS (Murashige and Skoog) growth medium containing 0.8% agar (Method S1), which was sealed with parafilm. The aseptic seedlings were cultivated in a climate room in Beijing, China, under 23–25 °C and a 16 h light/8 h dark photoperiod to collect all types of explants, excluding runner tips. 

### 4.2. Explants Preparation

Seeds and different vegetative parts of the strawberry plant (leaf discs, blended leaves, petioles, blended runner tips, runner tip meristematic tissues, and crowns) were used as explants in this research (Figure S1). The seeds were sterilized as described above. Leaf discs, petioles, and crowns taken from the two-month aseptic seedlings were cut into fragments. Runner tips from mature strawberry plants grown in the greenhouse were cut into pieces and sterilized by immersing them in 100% disinfectant (LIRCON, China; chlorine content 4.3 ± 0.6%) for 5 min, followed by repeat rinsing in sterile distilled water 5 times. Runner tip meristematic tissues were taken from the sterilized runner tips using a microscope. Blended leaves and blended runner tips were obtained using a sterilized blender. For each callus induction treatment using plant growth regulators, 90 pieces of explants were used.

### 4.3. Vector Construction and Agrobacterium-Mediated Transformation

**Figure 4:**
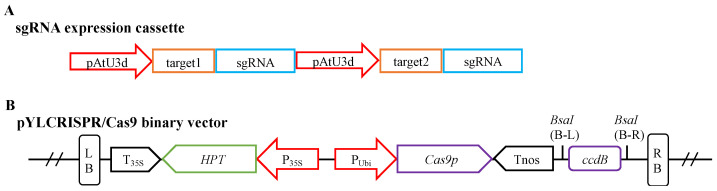
Block structures of the sgRNA expression cassette and the pYLCRISPR/Cas9 binary vector. (**A**) The structure of the sgRNA expression cassette. (**B**) The structure of the pYLCRISPR/Cas9 binary vector. pAtU3d, U3 small nuclear RNA promoter region from Arabidopsis; P_35S_, the promoter region of cauliflower mosaic virus 35S; P_Ubi_, the promoter region of ubiquitin gene from maize; T_35S_, 3^0^-termination signal of cauliflower mosaic virus 35S; T_nos_, 3^0^-termination signal of nopaline synthase gene; Cas9p: plant codon-optimized Cas9 gene; ccdB, a bacterial toxin that poisons DNA gyrase (as a negative selectable marker in the vector); HPT, coding region of hygromycin B phosphotransferase; LB and RB, right and left borders of T-DNA; BsaI (B-L and B-R), sites of restriction endonucleases BsaI.

The *FvMLO* gene family members *Fvb7-1* and *Fvb7-2* were identified as the homolog genes of the *AtMLO* genes in *Fragaria* × *ananassa*, which are associated with sensitivity to powdery mildew. The sequences of the two genes were obtained from the Genome Database for Rosaceae (GDR database; https://www.rosaceae.org/, accessed on 1 April 2021). To edit the two FvMLO genes simultaneously, the CRISPR-GE tool (http://skl.scau.edu.cn/, accessed on 1 April 2021) was used to access the target design (GGTGCACCGCTTGTTCAACC; CCAAGTACTCCATGCAAAGA). A single-guide RNA (sgRNA) expression cassette was designed and assembled for CRISPR/Cas9 gene editing, consisting of two modules, each containing an Arabidopsis snRNA promoter pAtU3d, a target sequence, and an sgRNA (Figure 4A; for primers, see Table S6). Each of the three fragments carried a *Bsa*I enzymatic cleavage site, which was used to assemble the sgRNA expression cassette into the pYLCRISPR/Cas9 vector (Figure 4B) using the Golden Gate cloning method [75] to complete the final vector construction [76]. The vector was verified by sequencing, and it was then transformed into *Agrobacterium tumefaciens* strain GV3101 for strawberry transformation.

### 4.4. Tissue Culture

The basal medium used in strawberry tissue culture consisted of MS salts, 30 g/L sucrose, and B5 vitamin and was solidified with 3.5 g/L plant phytagel (Method S1). The medium was sterilized at 121 °C for 15 min. Plant growth regulators and hormones (NAA, IAA, IBA, 6-BA, KT, TDZ, GA3, 2,4-D, PVPP) were added to the sterile medium individually or in various combinations with different concentrations for evaluating the effectiveness of callus induction, shoot regeneration, and rooting (Tables S1–S4). Each hormone was prepared in sterile water or KOH solution, following the specific storage requirements, and stored under −20 °C conditions.

The sterilized explants were placed onto the selection medium containing different hormone treatments. The medium was sealed with parafilm and moved to an incubator set at a temperature of 22 °C. The resistant callus obtained from the selection medium was transferred to the shoot induction medium under a 16 h photoperiod at a temperature range of 23–25 °C. Once the shoots were generated, they were transferred to the shoot elongation medium and incubated. When roots started to develop, the plantlets were moved to a medium suitable for strong seedling cultivation. Well-developed plantlets were subsequently planted in a climate room under the same growth environment conditions as the T_0_ plants.

The transformation efficiency of the strawberry was accessed and calculated in three separate parts as follows: the callus induction efficiency (%) of a certain explant was calculated by dividing the number of induced callus by the total number of explant pieces for that specific explant; the shoot induction efficiency (%) of a certain explant was calculated by dividing the number of induced shoots by the total number of calli obtained from that certain explant; and the shoot elongation rate (%) of a certain explant was calculated by dividing the number of elongated shoots by the total number of induced shoots under a specific hormone combination for that particular explant.

### 4.5. DNA Extraction, PCR Genotyping, and Sequencing

Leaf samples were harvested from T_0_ plants, and genomic DNA was extracted using a standard cetyl-trimethyl-ammonium bromide (CTAB) protocol [77]. The presence of Cas9 was verified using the primers of Cas9p-F and Cas9p-R. The Cas9-positive plants were then sequenced based on the Hi-TOM platform [78] to detect the engineered sequences within the target regions of the four *FvMLO* genes (*Fvb7-1*, *Fvb7-2*, *Fvb7-3*, and *Fvb7-4*). The library construction for Hi-TOM sequencing included two rounds of PCR (polymerase chain reaction). In the first round of PCR, target-specific sequences were amplified using the primers of 1Fvb7-F and 1Fvb7-R, and the products were further used as templates for the second round of PCR. The primers of 2Fvb7-F and 2Fvb7-R were used to amplify *Fvb7-1*, *Fvb7-2*, and *Fvb7-3* genes, while the primers of 2Fvb7-4F and 2Fvb7-R were used for amplifying the *Fvb7-4* gene. All the primers are listed in Table S6. After the second round of PCR, the amplicons were sent for NGS.

## 5. Conclusions

This report provides a protocol for the tissue culture of CRISPR/Cas9 gene-editing transformation in the strawberry cultivar Benihope (*Fragaria* × *ananassa* Duch cv. Benihope). The protocol recommends the use of leaf explants as the optimal choice for inducing callus tissue. The effectiveness of TDZ in inducing callus tissue was also evaluated, showing successful results in the nutrient medium, although the induction rate of callus tissue was slightly lower than that observed with NAA and 6-BA treatment. Additionally, the addition of PVPP to the shoot regeneration medium was proven to effectively suppress tissue browning and cell death, thereby facilitating the acquisition of transgenic plants. Given that the CRISPR/Cas9 gene-editing system is used for transgenic transformation, this protocol can be applied to the tissue culture of CRISPR/Cas9 gene-edited strawberries and is expected to be valuable for genetic editing breeding in strawberries.

## Figures and Tables

**Table 1:** The impact of different plant growth regulators on callus induction from leaf explants of strawberry (*Fragaria* × *ananassa* Duch. cv. Benihope).

Plant Growth Regulator (mg/L)					Callus Induction (%) ^a^
6-BA	NAA	TDZ	2,4-D	IBA	
0.5	0.5				44.3 ± 2.5
0.5	0.75				52.6 ± 2.1
**0.5**	**1**				**90.0 ± 5.0**
0.5	1.5				81.0 ± 6.0
		0.5	0.1		66.6 ± 2.9
		0.5	0.25		87.3 ± 2.5
		0.5	0.5		31.8 ± 4.6
		0.5		0.1	22.6 ± 2.5
		0.5		0.25	5.3 ± 1.5
		1		0.1	16.0 ± 2.0

^a^ Each value represents mean ± SD (*n* = 3 plates, each plate contained 30 pieces of explants). Abbreviations: 2,4-D: 2,4-dichlorophenoxyacetic acid; 6-BA: 6-benzylaminopurine; IBA: indolyl-3-butyric acid; NAA: 1-napthalene acetic acid; TDZ: thidiazuron.

**Table 2:** The impact of different plant growth regulators on callus induction from various explants of strawberry (*Fragaria* × *ananassa* Duch. cv. Benihope).

Plant Growth Regulator Concentration (mg/L)					Callus Induction from Different Explants (%) ^a^						
6-BA	NAA	TDZ	2,4-D	KT	Leaf Strips	Blended Leaf	Petiole	Blended Runner	Runner Tip Meristematic Tissue	Crown	Seed
0.5	0.5				44.3 ± 2.5	36.0 ± 2.0	38.6 ± 1.5	0	0	0	0
0.5	0.75				52.6 ± 2.5	44.0 ± 3.1	45.6 ± 2.1	0	0	0	0
**0.5**	**1**				**90.0 ± 5.0**	**82.5 ± 4.6**	**88.3 ± 4.5**	25.0 ± 2.0	31.3 ± 3.0	40.0 ± 2.5	0
0.5	1.5				81.0 ± 6.0	60.0 ± 5.0	66.3 ± 3.2	40.0 ± 3.0	44.6 ± 2.5	58.3 ± 5.5	0
		0.5	0.1		66.6 ± 2.9	56.6 ± 7.1	49.0 ± 5.6	59.8 ± 0.3	65.0 ± 5.0	55.0 ± 4.7	0
		**0.5**	**0.25**		87.3 ± 2.5	79.3 ± 6.1	64.0 ± 2.6	**86.0 ± 7.9**	**72.3 ± 2.5**	**87.3 ± 3.5**	**0**
		0.5	0.5		31.8 ± 4.6	20.3 ± 2.1	25.3 ± 2.9	41.6 ± 3.8	47.0 ± 2.6	45.2 ± 4.8	0
			1	0.5	0	0	0	0	0	0	49.0 ± 2.6
			2	0.5	0	0	0	0	0	0	65.1 ± 4.2
			3	0.5	0	0	0	0	0	0	34.3 ± 2.1

^a^ Each value represents mean ± SD (*n* = 3 plates, each plate contained 30 pieces of explants). Abbreviations: 2,4-D: 2,4-dichlorophenoxyacetic acid; 6-BA: 6-benzylaminopurine; NAA: 1-naphthalene acetic acid; TDZ: thidiazuron; KT: kinetin.

**Table 3:** The impact of different plant growth regulators on shoot elongation.

Plant Growth Regulators (mg/L)			Shoot Elongation Rate (%) ^a^	
GA3	6-BA	IBA	Segments of Leaf Explant	Runner Tip Meristematic Tissue Explant
3			0	0
2			0	0
1			0	0
	1	0.2	0	0
	0.5	0.2	0	0
	**0.25**	**0.2**	**50.0 ± 1.2**	**23.0 ± 0.6**

^a^ Each value represents mean ± SD (*n* = 5, each plate contained 10 samples). Abbreviations: 6-BA: 6-benzylaminopurine; GA3: gibberelic acid; IBA: indolyl-3-butyric acid.

## Data Availability

Data are contained within the article.

## Supplementary Materials

The following supporting information can be downloaded at https://www.mdpi.com/article/10.3390/plants13050563/s1: Method S1: The nutrient concentrations of plant tissue culture basal media used in this research, Figure S1: Preparation of explants, Figure S2: Callus induced from different explants, Figure S3: *Agrobacterium*-transformation steps of the strawberry through different explants, Table S1: Different concentrations and combinations of plant growth regulators added to the callus induction medium to evaluate their effects on callus induction efficiency from leaf strip explants from strawberry (*Fragaria* × *ananassa* Duch. cv. Benihope), Table S2: Different concentrations and combinations of plant growth regulators added to the callus induction medium for evaluating callus induction efficiency from various explants of blended leaves, petioles, runner tips, runner tip meristematic tissues, crowns, and seeds from strawberry (*Fragaria* × *ananassa* Duch. cv. Benihope), Table S3: Different concentrations and combinations of plant growth regulators added to the shoot induction medium to evaluate their effect on shoot induction efficiency from calli produced by different explants, Table S4: Different concentrations and combinations of plant growth regulators added to the shoot elongation medium, Table S5: Sequencing and genotyping of the Cas9-positive strawberry plants, Table S6: Primers designed in this research, Data S1: Raw data of Hi-TOM sequencing (No. USR-14596), Data S2: Raw data of Hi-TOM sequencing (No. USR-15933), Data S3: Reference genome of the wild-type strawberry used for genotyping.
