# Supplementary material for: An Efficient Agrobacterium-Mediated Genetic Transformation System for Gene Editing in Strawberry (Fragaria × ananassa)
Source: Plants (Basel). 2024 Feb 20;13(5):563. doi: 10.3390/plants13050563 (PMC10934387; doi:10.3390/plants13050563)
Supplement: Supplementary file 1 [file plants-13-00563-s001.zip › plants-2757540_supplementary.pdf]

Method S1. The nutrient concentrations of plant tissue culture basal media used in this research.
Figure S1. Preparation of explants.
Figure S2. Callus induced from different explants.
Figure S3. Agrobacterium-transformation steps of the strawberry through different explants.
Table S1. Different concentrations and combinations of plant growth regulators added to the callus induction medium to evaluate their effects on callus induction efficiency from leaf strips explants from strawberry (*Fragaria* x *ananassa* Duch. cv. Benihope).
Table S2. Different concentrations and combinations of plant growth regulators added to callus induction for evaluating callus induction efficiency from various explants of blended leaves, petioles, runner tips, runner tip meristematic tissues, crowns, and seeds from strawberry (*Fragaria* x *ananassa* Duch. cv. Benihope).
Table S3. Different concentrations and combinations of plant growth regulators added to the shoot induction medium to evaluate their effect on shoot induction efficiency from callus produced by different explants.
Table S4. Different concentrations and combinations of plant growth regulators added to the shoot elongation medium.
Table S5. Sequencing and genotyping of the Cas9-positive strawberry plants.
Table S6. Primers designed in this research.
Data S1. Raw data of Hi-TOM sequencing (NO. USR-14596).
Data S2. Raw data of Hi-TOM sequencing (NO. USR-15933).
Data S3. Reference genome of the wildtype strawberry used for genotyping.

Method S1 The nutrient concentrations of plant tissue culture basal media used in this research.

1. **Agrobacterium-infection medium:**
   MS salts + B5 vitamins + sucrose (30 g/L) + glucose (10 g/L) + Fe^{3+} (5 ml/L) + MES (0.5 g/L) + AS (40 mg/L); pH = 5.5.
2. **Co-cultivation medium:**
   MS salts + B5 vitamins + sucrose (30 g/L) + glucose (10 g/L) +MES (0.5 g/L) + Fe^{3+} (5 ml/L) + AS (40 mg/L) + Phytagel (3.5 g/L); pH = 5.5.
3. **Callus-induction medium:**
   MS salts + B5 vitamins + sucrose (30 g/L) + Fe^{3+} (5 ml/L) + MES (0.5 g/L) + different concentrations and combinations of growth regulators (Supplementary Table S2) + Phytagel (3.5 g/L); pH = 5.8; Timentin (250 mg/L) was added after autoclaving.
4. **Selection medium:**
   MS salts + B5 vitamins + sucrose (30 g/L) + Fe^{3+} (5 ml/L) + MES (0.5 g/L) + different combinations and concentrations of growth regulators (Supplementary Table S2) + Phytagel (3.5 g/L); pH = 5.8; Timentin (250 mg/L) and Hygromycin (4 mg/L) were added after autoclaving.
5. **Shoot-induction Medium:**
   MS salts + B5 vitamins + sucrose (30 g/L) + Fe^{3+} (5 ml/L) + different concentrations and combinations of growth regulators (Supplementary Table S3) + Phytagel (3.5 g/L); pH = 5.8; Timentin (250 mg/L) and Hygromycin (4 mg/L) were added after autoclaving.
6. **Shoot-elongation Medium:**
   MS salts + B5 vitamins + sucrose (30 g/L) + Fe^{3+} (5 ml/L) + different concentrations and combinations of growth regulators (Supplementary Table S4) + Phytagel (3.5 g/L); pH = 5.8; Timentin (250 mg/L) and Hygromycin (4 mg/L) added after autoclaving.
7. **Seedling-strengthen medium:**
   MS salts (full and half) + B5 vitamins (1 ml/L) + sucrose (30 g/L) + Fe^{3+} 5ml/L + IBA (0.1 mg/L) + Agar (6 g/L); pH = 5.8; Timentin (250 mg/L) and Hygromycin (4 mg/L) added after autoclaving.
8. **Seed-germination medium**:
   MS salts (half) + B5 vitamins (1 ml/L) + sucrose (15 g/L) + Fe^{3+} (5 ml/L) + Agar (5 g/L); pH = 5.8.

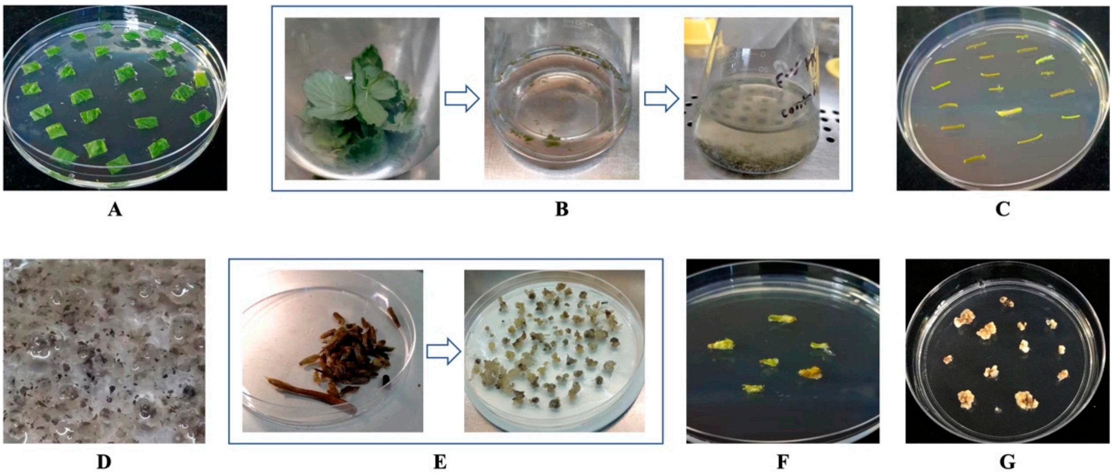

Figure S1 Preparation of explants. A, leaf strips; **B**, the blended leaves; **C**, segments of petioles; **D**, blended runner tips; **E**, runner tip meristematic tissues; **F**, callus induced from runner tip meristematic tissues; **G**, small pieces of crowns; **H**, callus induced from seeds.

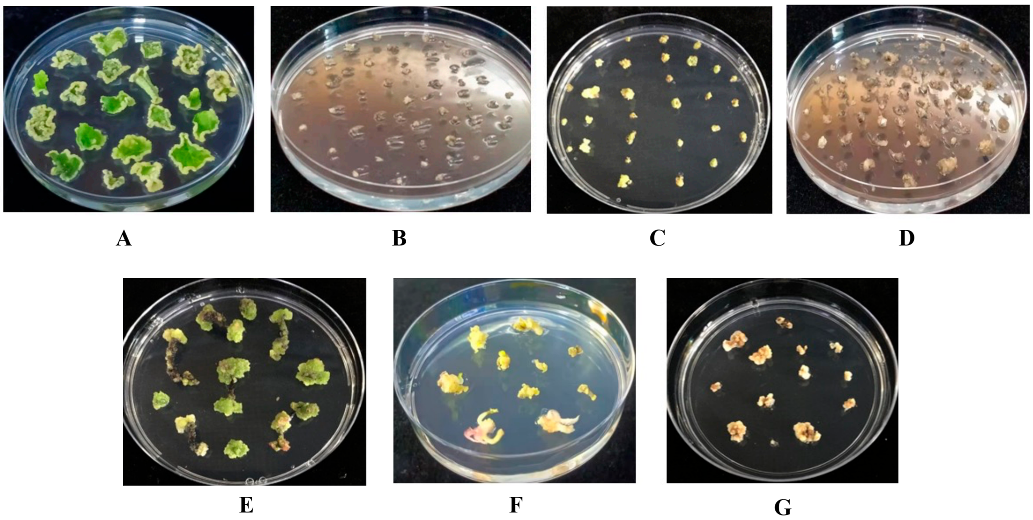

Figure S2 Callus induced from different explants. Callus induced from **A**, leaves; **B**, blended leaves; **C**, blended runner tips; **D**, runner tip meristematic tissues; **E**, petioles; **F**, crowns; **G**, seeds.

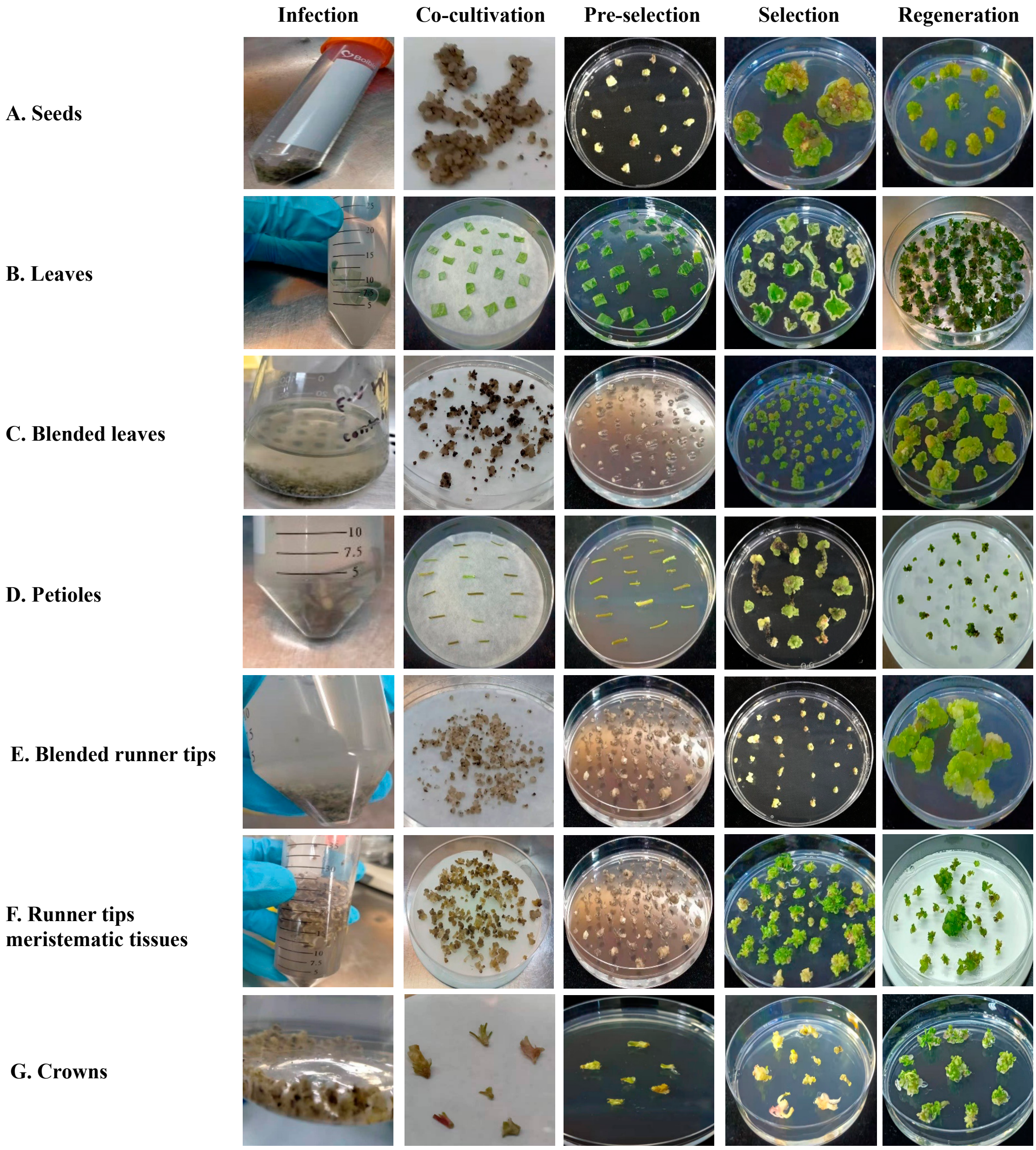

Figure S3 Agrobacterium-transformation steps of the strawberry through different explants. A-G represent the tissue culture steps of Agrobacterium-mediated transformation using different types of explants. Seeds, blended leaves, petioles, and blended runner tips failed to regenerate any shoot under all treatments.

Table S1 Different concentrations and combinations of plant growth regulators added to the callus induction medium to evaluate their effects on callus induction efficiency from leaf strips explants from strawberry (*Fragaria* x *ananassa* Duch. cv. Benihope).

Treat Nr.	Plant growth regulator concentration (mg/L)				
	6-BA	NAA	TDZ	2,4-D	IBA
1	0.5	0.5	0	0	0
2	0.5	0.75	0	0	0
3	0.5	1	0	0	0
4	0.5	1.5	0	0	0
5	1	0.5	0	0	0
6	1	0.75	0	0	0
7	1	1	0	0	0
8	1	1.5	0	0	0
9	1.5	0.5	0	0	0
10	1.5	0.75	0	0	0
11	1.5	1	0	0	0
12	1.5	1.5	0	0	0
13	0	0	0.5	0.1	0
14	0	0	0.5	0.25	0
15	0	0	0.5	0.5	0
16	0	0	1	0.1	0
17	0	0	1	0.25	0
18	0	0	1	0.5	0
19	0	0	1.5	0.1	0
20	0	0	1.5	0.25	0
21	0	0	1.5	0.5	0
22	0	0	0.5	0	0.1
23	0	0	0.5	0	0.25
24	0	0	0.5	0	0.5
25	0	0	1	0	0.1
26	0	0	1	0	0.25
27	0	0	1	0	0.5
28	0	0	1.5	0	0.1
29	0	0	1.5	0	0.25
30	0	0	1.5	0	0.5

Table S2 Different concentrations and combinations of plant growth regulators added to callus induction for evaluating callus induction efficiency from various explants of blended leaves, petioles, runner tips, runner tip meristematic tissues, crowns, and seeds from strawberry (*Fragaria* x *ananassa* Duch. cv. Benihope).

Treat Nr.	Plant growth regulator concentration (mg/L)				
	6-BA	NAA	TDZ	2,4-D	KT
1	0.5	0.5	0	0	0
2	0.5	0.75	0	0	0
3	0.5	1	0	0	0
4	0.5	1.5	0	0	0
5	0	0	0.5	0.1	0
6	0	0	0.5	0.25	0
7	0	0	0.5	0.5	0
8	0	0	0	1	0.5
9	0	0	0	2	0.5
10	0	0	0	3	0.5

Table S3 Different concentrations and combinations of plant growth regulators added to the shoot induction medium to evaluate their effect on shoot induction efficiency from callus produced by different explants.

	Supplemented plant growth regulators (mg/L)					
Treat Nr.	6-BA	NAA	TDZ	2,4-D	IBA	IAA
1	1	0	0	0	0	0
2	2	0	0	0	0	0
3	3	0	0	0	0	0
4	1	0.1	0	0	0	0
5	2	0.1	0	0	0	0
6	3	0.1	0	0	0	0
7	1	0.2	0	0	0	0
8	2	0.2	0	0	0	0
9	3	0.2	0	0	0	0
10	1	0	0	0.1	0	0
11	2	0	0	0.1	0	0
12	3	0	0	0.1	0	0
13	1	0	0	0.2	0	0
14	2	0	0	0.2	0	0
15	3	0	0	0.2	0	0
16	1	0	0	0	0.1	0
17	2	0	0	0	0.1	0
18	3	0	0	0	0.1	0
19	1	0	0	0	0.2	0
20	2	0	0	0	0.2	0
21	3	0	0	0	0.2	0
22	1	0	0	0	0	0.1
23	2	0	0	0	0	0.1
24	3	0	0	0	0	0.1
25	0	0	1	0	0	0
26	0	0	2	0	0	0
27	0	0	1	0.1	0	0
28	0	0	2	0.1	0	0
29	0	0	1	0	0.1	0
30	0	0	2	0	0.1	0
31	0	0	1	0	0	0.1
32	0	0	2	0	0	0.1
33	0.5	0.1	0	0	0	0
34	0.5	0	0	0.1	0	0
35	0.5	0	0	0	0.1	0
36	0.5	0	0	0	0	0.1
37	0	0	0.5	0.1	0	0
38	0	0	0.5	0	0.1	0

Table S4 Different concentrations and combinations of plant growth regulators added to the shoot elongation medium.

	Supplemented plant growth regulators			
Treat Nr.	GA3 (mg/L)	6-BA (mg/L)	IBA (mg/L)	PVPP (g/L)
1	3	0	0	2
2	2	0	0	2
3	1	0	0	2
4	0	0.25	0.2	2
5	0	0.5	0.2	2
6	0	1	0.2	2

Table S6 Primers designed in this research.

Primer	Sequence
Vector construction:	
pAtU3d-f	TTCAGAGGTCTCTACCGATAAGCTTATGATTTCTTTT
pAtU3d-r	AGCGTGGGTCTCGCACCTGACCAATGGTGCTTTGTA
sgRNA-pAtU3d-f	TTCAGAGGTCTCTGGTGCACCGCTTGTTCAACCGTTTCAGAGCTAGAAATAGCAAG
sgRNA-pAtU3d-r	AGCGTGGGTCTCGTTGGTGACCAATGGTGCTTTGTA
sgRNA-f	TTCAGAGGTCTCTCCAAGTACTCCATGCAAAGAGTTTCAGAGCTAGAAATAGCAAG
sgRNA-r	AGCGTGGGTCTCGCTCG CCATCCACTCCAAGCTCTT
Hi-TOM sequencing:	
1Fvb7-F	CAGTGCTGTTCCTGTTGTCTA
1Fvb7-R	CATGCAAAGAAGGCCAGCTGA
2Fvb7-F	GGAGTGAGTACGGTGTGCGGTGGGAACTAAACTACAAGT
2Fvb7-R	GAGTTGGATGCTGGATGGCTAGCAAGTTGAACTACCTGGA
2Fvb7-4F	GAGTTGGATGCTGGATGGCTACCTGGAATAAAGCAAAATGG
Cas9 identification:	
Cas9p-F	GATCCTTTCCGCTAGGCTCT
Cas9p-R	CTCCACCGTCAATGTAACCG
